# Possible Influence of Natural Events on Heavy Metals Exposure from Shellfish Consumption: A Case Study in the North-East of Italy

**DOI:** 10.3389/fpubh.2015.00021

**Published:** 2015-02-04

**Authors:** Carmen Losasso, Laura Bille, Ilaria Patuzzi, Monica Lorenzetto, Giovanni Binato, Manuela Dalla Pozza, Nicola Ferrè, Antonia Ricci

**Affiliations:** ^1^Department of Food Safety, Istituto Zooprofilattico Sperimentale delle Venezie, Legnaro, Italy; ^2^Laboratory of Epidemiology of Water Environment, Istituto Zooprofilattico Sperimentale delle Venezie, Legnaro, Italy; ^3^GIS Unit, Istituto Zooprofilattico Sperimentale delle Venezie, Legnaro, Italy; ^4^Laboratory of Chemistry, Istituto Zooprofilattico Sperimentale delle Venezie, Legnaro, Italy

**Keywords:** cadmium, shellfish, food safety, flooding, exposure

## Abstract

The objective of this study was the estimation of the exposure over time to heavy metals (cadmium, mercury, and lead) due to shellfish consumption in the Veneto Region, Italy. Shellfish consumption was investigated by a food frequency consumption survey. Altogether, 1949 households, stratified into the five most populated areas of the Veneto Region, were involved in the study. Exposure estimation to heavy metals was carried out taking into account the level of metal measured in samples of Manila clams (*Ruditapes philippinarum*) and grooved carpet shell (*Ruditapes decussatus*), collected in the frame of the monitoring activities of mollusk production areas of Veneto Region, between January 2007 and December 2012. A general high contribution of the considered shellfish to the Tolerable Weekly Intake was noticed in the case of cadmium, especially in 2011, when a considerable increase in cadmium intake was estimated. This was probably due to a heavy rainfall event that triggered catastrophic flooding with high impact on shellfish capture areas in November 2010. The results strongly emphasize the importance of dealing with food safety in a holistic way, taking into account the potential impact of extraordinary natural events on food chain contamination, in order to identify food hazards at an early stage, before developing into a real risk for consumers.

## Introduction

Seafood has been acknowledged as an integral component of a well balanced diet, providing a healthy source of energy, high quality proteins, and a wide range of other important nutrients (1, 2). In contrast to the potential health benefits of dietary seafood intake, the chemical pollutants contained in these products have emerged as an issue of concern, particularly for frequent consumers (3–6).

In this regard, heavy metal contamination is recognized as a public health hazard because of the widespread diffusion of these compounds in the environment, including the marine ecosystem (7). Heavy metals can be accumulated by marine organisms due to their presence in water and sediments or in the marine food chain (8, 9). Thus, diets containing seafood represent the main route of exposure to these elements in the general human population (3).

Heavy metals in aquatic systems can be naturally produced by leaching from soil/rock to water, which usually produces low levels, causing no serious deleterious effects on human health (10). However, the pollution of marine environments is mainly due to the development of human activities that result in direct or indirect chemical release into the aquatic environment. In particular, it depends on the contaminant loads carried into the sea by rivers and other watercourse basins, which drain areas of intense urbanization, and on the direct input of municipal and industrial waste (11).

Some heavy metals may transform into persistent metallic compounds with high toxicity (12), which can be bioaccumulated in organisms, concentrating in the food chain, and thus threatening human health (12). The toxicity of mercury, for example, is proportional to its degree of organization, which makes it more available for biota (13). In this context, biomonitoring based on sampling and analysis of seafood can provide direct evidence of alterations occurring in the ecosystem due to environmental pollution (12).

Delivered contaminant loads can display a temporal variability on the overall pollutant transfers to marine environments (14, 15) due to specific and time limited catastrophic events such as calamitous flooding (16). In these cases, significant concerns exist regarding the potential toxic hazards for food safety, due to the increase of chemical contaminants levels in the marine environment with a consequent enhancement of pollutant accumulation in seafood (17).

Measured concentrations of total heavy metals generally correlate with amounts of suspended particulate matter, due to the preferred association of metals with fine materials suspended in the water column (18). Thus, the load of heavy metals increases in relation to both discharge and suspended sediment transport.

A number of studies have demonstrated the role of flood events in the delivery of chemical contaminants to the Venice lagoon from rivers (18–21). For some streams, the response in terms of transport is strong, particularly for suspended particulate matter and total heavy metals, and it has been estimated that one single day of flood can equal the monthly load in normal flow conditions (18). Moreover, the short time period that characterizes the load delivery in floods could lead to potentially harmful effects in the receiving lagoon ecosystem.

A global-scale monitoring program based on the “sentinel organism concept” has been outlined that is capable of detecting trends in concentrations of several marine contaminants. Marine shellfish, being filter-feeding organisms known to accumulate heavy metals, have proven to be ideal candidates (12, 22). Several attributes make shellfish superior to other organisms for environmental monitoring, including their wide geographical distribution and abundance in stable populations, their sedentary habits, tolerance to environmental fluctuations, and to various environmental contaminants, their very low-level metabolism of organic contaminants, plus their being reasonably long-lived and of suitable size (12).

The purpose of this study was to estimate the exposure over time to heavy metals due to shellfish consumption in the Veneto Region. The possible concerns for food safety are discussed in the light of a recent catastrophic flooding event that involved the north-east of Italy at the end of 2010.

## Materials and Methods

### Shellfish consumption survey

Between December 2010 and May 2012, a food consumption survey was conducted on 1949 households stratified into the five most populated areas of the Veneto Region (north-east of Italy) corresponding to the provinces of Treviso, Padua, Verona, Vicenza, and Venice. One healthy individual aged between 14 and 92 years, per household, voluntarily participated in the food consumption survey; a total of 1355 females and 594 males responded in the study.

The questionnaire was divided into two main sections (23), and is available on request to the authors. In the first part, information on respondents’ characteristics (age, gender, involvement in food purchasing, and cooking) was collected; in the second part (17 items), called “*nutritional safety*,” information was collected on the consumption frequency of a number of food items, among which were shellfish and fish. Participants were asked to answer the questions according to their specific habits and to specify the size of their shellfish servings by comparison with images of three different serving sizes. Participants filled in the questionnaire autonomously, and self-reported responses were later entered into an electronic database (Access 2009, Microsoft Corporation, Redmond, WA, USA). Each entry was validated comparing the original questionnaires and the database records. Categorical data were summarized as counts with percentages and continuous data as averages with their relative standard deviation (SD). The study was conducted according to the guidelines laid down in the Declaration of Helsinki; written informed consent was obtained from all participants.

### Exposure assessment

The exposure estimation over time was carried out taking into account the concentration of cadmium (Cd) measured in samples of Manila clams (*Ruditapes philippinarum*) and grooved carpet shell (*Ruditapes decussatus*) reared in the Venice lagoon and coastal areas of the Veneto Region, North-eastern Italy. These two species were chosen as representative of the lagoon’s shellfish production and as significant in local dietary habits. Moreover, the Veneto region is the primary national producer of Manila clams and Italy is the leading European producer of this species.

### Sample collection

Shellfish sampling was performed between January 2007 and December 2012 in the framework of the classification and monitoring activities of mollusk production areas of Veneto region (North-Eastern Mediterranean Italian coast, Adriatic Sea), in order to determine levels of cadmium, mercury, and lead (Cd, Hg, and Pb), as previously reported (24).

The number of shellfish samples analyzed from 2007 to 2012 in order to check Cd, Hg, and Pb contamination levels are described in Table 2.

### Experimental analysis

Cd and Pb concentrations were determined by means of Electrothermal Atomic Absorption Spectrometry (ETAAS) using an M6 mkII Atomic Absorption Spectrometer (Thermo Electron, Cambridge, UK) with D2 and Zeeman background correction, equipped with a GF95 Graphite Furnace atomizer (Table 1). For Hg determination, a Thermal Decomposition Amalgamation and Atomic Absorption Spectrophotometry (TDA AAS) direct analyzer FKV AMA254 (Altec Ltd., Prague, CZ) was employed.

**Table 1:** **Instrumental parameters for GFAAS determination**.

Parameter	Pb	Cd
Wavelength (nm)	283.3	228.8
Slit (nm)	0.5	0.5
Measurement time (s)	3.0	3.0
Background correction	D_2_	D_2_
Atomization (t°C)	1500	1300

Analytical methods details were previously described (24).

### Intake estimation

Metal concentrations were expressed in milligrams per kilogram of fresh weight of shellfish (mg/kg).

The Estimated Weekly Intake (EWI) was determined based on the average concentration of metal in shellfish tissue and the average weekly shellfish consumption rate, as declared by survey respondents.

The EWI for adults was calculated as follows:

$$\mathrm{EWI}=\left(C_{m}\times IR_{w}\right)∕\mathrm{BW}$$

where C_m_ is the average metal concentration in shellfish expressed as milligrams per kilogram of fresh weight, IR_w_ represents the weekly average consumption of shellfish estimated as 150 g based on the dietary intake survey, and BW is the body weight of a hypothetical adult of 70 kg (25).

The health risk of Cd ingestion *via* consumption of shellfish was assessed based on the target hazard quotient (THQ). The THQ is a ratio of determined dose of a pollutant to a reference dose level. If the ratio is <1, the exposed population is unlikely to experience obvious adverse effects. The method of estimating risk using THQ was provided in the US EPA Region III risk based concentration table (26) and it is described by the following equation:

THQ=(EFr×ED×FI×MC∕RfD×BW×AT)×0.001

where THQ is target hazard quotient; EFr is exposure frequency (365 days/year); ED is the exposure duration; FI is shellfish ingestion (21.43 g/person/day); MC is Cd average concentration in shellfish; RfD is the Cd oral reference dose (0.001 lg/g/day) (27, 28); BW is the average body weight for an adult male (70 kg); and AT is the averaging time for non-carcinogens (365 days/year × number of exposure years).

In this study, the risk was estimated for the intake of only one toxicant (Cd) *via* one food item (shellfish). Thus the equation was simplified as follows:

THQ=(FI×MC∕RfD×BW)×0.001

### Shellfish sampling area mapping

Shellfish production areas were used to locate the analyzed shellfish. Cd concentration averages were calculated for each year (2007–2012) and for each monitoring area in order to obtain six maps representing the yearly Cd spatial distribution (data not shown).

In order to compare Cd values observed, the irregularly shaped monitoring areas were rasterized with a cell size of 140 m × 140 m. The cell size corresponded to 1/4 of the smallest monitoring area surface (29).

Map algebra was applied to calculate the raster map of the difference between the average of Cd values for the six monitored years and the average values in the year 2011 (Figure 3).

The analysis was carried out using ESRI ArcInfo GIS software (Environmental Systems Resource Institute, ESRI^®^ ArcMapTM 10.0, Redlands, CA, USA)

## Results

### Cd, Hg, and Pb levels in shellfish

Cd, Hg, and Pb concentrations in the shellfish, expressed as milligrams of element per kilogram of fresh weight (mg/kg), are summarized in Table 2 where the descriptive statistics for annual means are reported.

**Table 2:** **Cd, Hg, and Pb concentrations expressed as milligrams of element per kilogram of fresh weight (mg/kg)**.

Year	*N*	Cd (mg/Kg)				Hg (mg/Kg)				Pb (mg/Kg)			
		Mean	Min	Max	SD	Mean	Min	Max	SD	Mean	Min	Max	SD
2007	164	0.0793	0.01	0.30	0.0528	0.0486	0.0019	0.17	0.0343	0.2405	0.02	1.18	0.1260
2008	162	0.0652	0.01	0.25	0.0408	0.0504	0.0100	0.19	0.0315	0.2338	0.04	0.89	0.1100
2009	196	0.0565	0.01	0.17	0.0343	0.0468	0.0100	0.13	0.0254	0.2249	0.05	0.64	0.1104
2010	167	0.0525	0.01	0.18	0.0325	0.0404	0.0100	0.11	0.0183	0.1576	0.03	0.51	0.0825
2011	136	0.0991	0.01	0.34	0.0616	0.0450	0.0019	0.12	0.0269	0.1801	0.04	0.49	0.0917
2012	122	0.0708	0.02	0.25	0.0485	0.0480	0.0100	0.18	0.0326	0.1588	0.03	0.38	0.0697

Heavy metal concentrations were largely below the maximum levels established by Regulation EC/1881/2006 (1, 0.5, and 1.5 mg/kg for Cd, Hg, and Pd, respectively) (30, 31). Moreover, among the three heavy metals, Pb clearly accumulated to the highest level in the shellfish (Table 2).

In addition, shellfish harvested in 2011 had higher average levels of Cd compared with those harvested in the other investigated years (Table 2). Similarly, during 2011, shellfish contained greater amounts of Pb than during the previous year (0.1801 and 0.1576 mg/kg in 2011 and 2010, respectively). On the contrary, mean annual Hg levels in the shellfish did not significantly vary.

**Figure 1:**
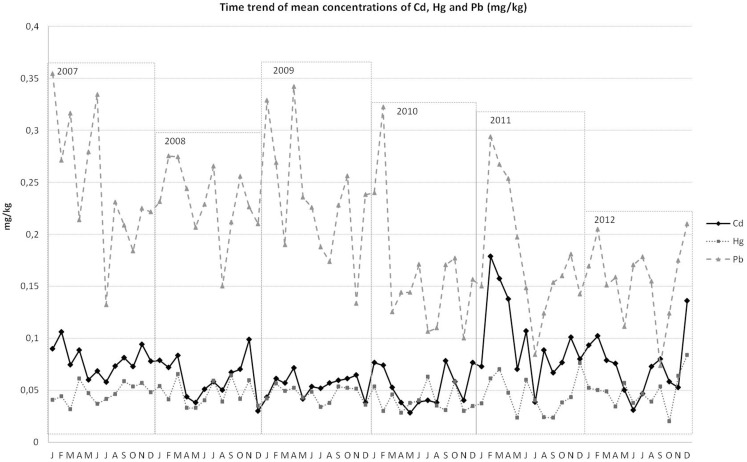
**Mean concentrations of Cd, Pb, and Hg (milligram per kilogram) over time**.

Figure 1 describes the concentrations of heavy metals in shellfish, measured monthly over time. Despite Pb and Hg levels being unstable over time and clearly undergoing seasonal variations, the range of these differences was constant over the investigated years. On the contrary, Cd levels severely increased between January and May 2011 (Figure 1).

### Estimates of Cd intake due to shellfish consumption

In the current study, as Cd levels in the shellfish had shown such an anomalous increase during early 2011 (see Cd, Hg, and Pb Levels in Shellfish), the exposition analyses were focused on determining the potential amounts of this heavy metal ingested by consumers, and the causes of that increase.

A food consumption survey was conducted to estimate the relative contribution of shellfish to the weekly Cd intake *via* food.

Demographic characteristics of the respondents are described in Table 3. More than half of the respondents were females (69.5); the median number of family members was 3.83. Respondents were subdivided into the following four age groups: group 1, from 10 to 18 years (*N* = 734), group 2, from 19 to 40 years (*N* = 248), group 3, from 41 to 65 years (*N* = 586), and group 4, from 66 to 94 years (*N* = 381).

**Table 3:** **Sample demographic description**.

Age class	10–18	19–40	41–65	66–94	Total
*N*	734	248	586	381	1949
Gender (female %)	51.2	86.3	81.6	75.3	69.5
Number of family members (mean)	4.04	4.10	3.68	3.25	3.83

**Figure 2:**
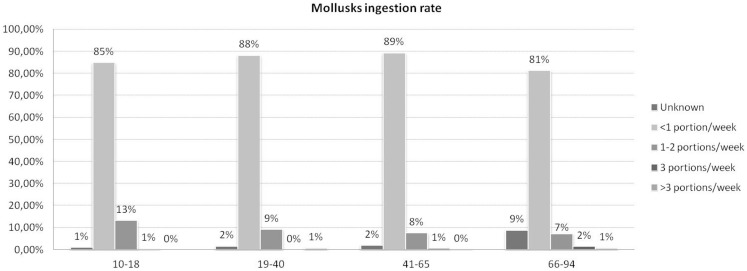
**Shellfish weekly consumption rates in the four indicated age groups (10–18; 19–40; 41–65; and 66–94)**.

As shown in Figure 2, the majority of respondents belonging to each age group consumed less than one serving of shellfish per week and the average size of the consumed shellfish servings was estimated to be 150 g, based on the respondents’ replies.

The EWI of Cd for an average person weighing 70 kg is reported in Table 4, taking into consideration the mean Cd concentrations determined in this study and shellfish consumption rates reported by respondents in the survey. The calculated intakes were further compared with the corresponding tolerable weekly intake (TWI), estimated to be 2.5 μg/kg of body weight (32). Even though the EWI values for Cd due to shellfish consumption were always below the TWI, and therefore the THQ levels remained below the risk value of 1, shellfish generally display a high contribution to the TWI for Cd. Indeed, as shown in Table 4, a dramatic increase of Cd intake due to shellfish consumption was estimated in 2011, compared with the previous year.

**Table 4:** **Estimated weekly intake (EWI) and tolerable weekly intake (TWI) for Cd expressed as microgram per kilogram of body weight**.

Year	EWI (mg/kg)	TWI (mg/kg)	% TWI	THQ	% Of Cd intake
2007	0.17	2.5	7	0.024	–
2008	0.14	2.5	6	0.020	−18
2009	0.12	2.5	5	0.017	−13
2010	0.11	2.5	5	0.016	−7
2011	0.21	2.5	8	0.030	89
2012	0.15	2.5	6	0.022	−29

*% TWI indicates the contribution in percentage of shellfish consumption to the TWI. % Of Cd intake indicates the variation in percentage of Cd intake over time due to shellfish consumption. The values indicate the variation compared with the contribution of the previous year. THQ refers to the total hazard quotient*.

### Shellfish sampling area mapping

**Figure 3:**
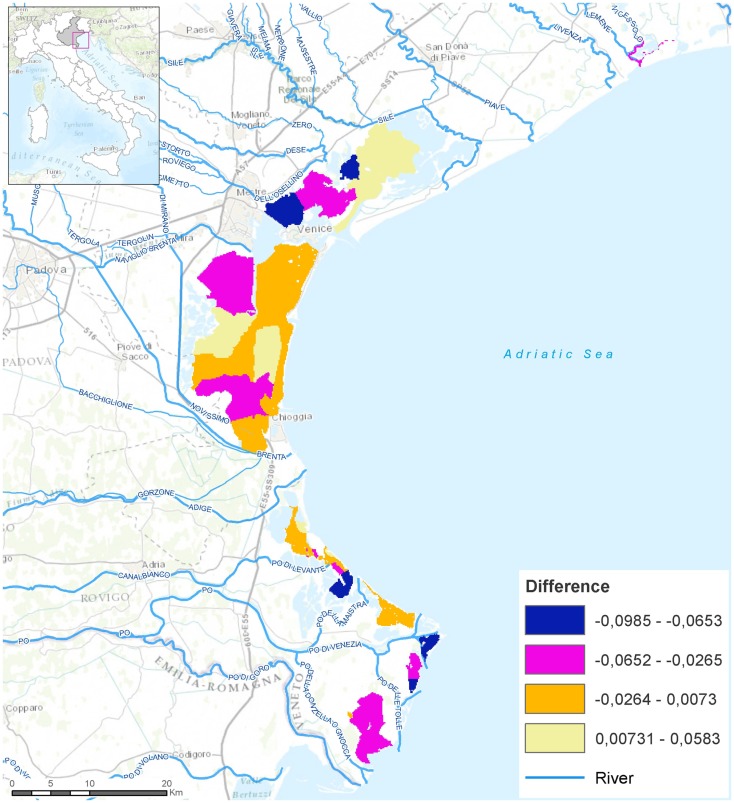
**Difference between the average of cadmium values for the 2007–2012 years and the year 2011**.

In order to identify the main areas of shellfish bioaccumulation of Cd, raster maps of the yearly spatial distribution and of the difference between the average value for the six monitored years and the average values recorded in 2011 (Figure 3), were analyzed.

The maps were portrayed according to the ESRI Natural Breaks classification method (33). In this classification method, data distribution is explicitly considered, within-class variance is minimized and between-class variance is maximized (34, 35).

Classes of negative values suggest that the average Cd levels in shellfish for the year 2011 were higher than the average values detected during the other 5 years.

As shown in Figure 3, the main areas where Cd was found to accumulate in shellfish were the north and the far south lagoon basins.

## Discussion

The aim of this study was the assessment of heavy metal exposure over time due to shellfish consumption in the Veneto Region focusing on three heavy metals (cadmium, mercury, and lead), as requested by the European Union regulation for hazardous metals (30).

Intake data were obtained from a food consumption survey that involved different areas spanning the Veneto Region, while shellfish contamination data were obtained via the classification and monitoring activities of mollusk production areas of Veneto region.

Nonetheless, in order to have a homogeneous sample size among years and species, and due to the shellfish consumption habits of the Veneto population, only Manila clams (*Ruditapes philippinarum*) and grooved carpet shell (*Ruditapes decussatus*) metal contamination data were included in the study.

Results obtained from heavy metal contamination monitoring activity show that among the three studied heavy metals, Pb accumulated to the highest levels in the investigated shellfish.

Generally, only low levels of all three heavy metals were detected in the shellfish. However, a significant difference in the heavy metal concentrations over time was noticed, especially in the case of Cd, which underwent a rapid increase in early 2011. This observation caused us to evaluate the impact of the rise of Cd concentration in the selected shellfish on consumers’ exposure.

The potential health risk to the local population from Cd intake *via* consumption of shellfish was assessed by comparing the EWI with the TWI and by estimating the THQ.

Results show that shellfish substantially contributed to the TWI for Cd, particularly during 2011, even though the EWI values were below the TWI in each of the sampled years and the THQ never reached the value 1. However, the two investigated shellfish species are not the only potential source of dietary Cd, since this metal can be ingested via many other food items comprising the diet of the target population (data not shown).

We speculate that a heavy rainfall event that triggered catastrophic flooding in the days leading up to November 3, 2010, with abundant rainfall affecting large areas of the Veneto Region, was likely to have been responsible for the increased Cd levels detected in early 2011.

The principal rivers involved in the flood event were the Bacchiglione river (140 sq km flooded containing a total of 380 Municipalities), and the Brenta river, two of the local tributaries of the southern basin of Venice lagoon, while an impact on the river basins of upper Piave, Sile, the drainage basin in the lagoon and the river Po also occurred. The contaminants accumulated in the sediment might have been mobilized by the effect of biological and physical mechanisms, including the hydrodynamics of the water exchange between the lagoon and the Adriatic Sea (20) and which then led to Cd accumulation in shellfish captured in the areas where these rivers flow, as indicated in Figure 3.

Regarding the northern part of the lagoon where high levels of Cd accumulation were found (Figure 3), apart from the input of the Piave and Sile rivers, other contaminated sources could have contributed to the increase of Cd load in 2011, such as discharge from surrounding heavily industrialized areas, pollution produced by the city of Venice and wastewater treatment plants (20).

Contaminant fate analyses, though, suggests that such contamination routes could not reach the far southern basin (data not shown). Here, Cd (and indeed, likely other heavy metal) loadings mostly originate from local tributaries (20) including the Bacchiglione river. Thus, only a small fraction of contaminants from the dominant loading sources in the central basin reach the inlet, through which most water export to the Adriatic Sea occurs. Naturally, this limits seaward transfer of the contaminants, trapping most of them in the sediment of the Venice lagoon (20), and making them available to be funneled into the human food chain *via* harvested shellfish consumption.

Moreover, according to the 2011 report on the environmental pollution in the Venetian Lagoon basin, published by the Agency for Water Control of the Veneto Region (36) a strict dependence of water quality delivered to the Venice lagoon from precipitation, in terms of presence of pollutants such as heavy metals, was detected.

In fact, the analytical determinations carried out on samples of first flushings from tanks proved the high level of pollution of the water drained off in the Venetian Lagoon during the spring and autumn months, when intense rainfalls occur.

Furthermore, the report strongly emphasized the critical contribution posed by the ring road surrounding the Venice area to heavy metal concentration in the drainage water, confirming the significant influence of road runoff to heavy metal flow into natural water bodies.

The observations made so far strongly emphasize the importance of approaching food safety in a holistic way, taking into account the potential impact of extraordinary natural events on food chain contamination.

An important objective is to provide for the activation of emergency monitoring able to identify potential increases in food hazards at an early stage and enabling hazard analysis to be conducted on time, before a real risk for consumers develops. A case in point is highlighted by the results of the current study, indicating that flood events on land could result in increased heavy metal contamination in edible mollusks at sea, potentially producing increased lead-on risks for heavy metal bioaccumulation in shellfish consumers.

## Conflict of Interest Statement

The authors declare that the research was conducted in the absence of any commercial or financial relationships that could be construed as a potential conflict of interest.
